# Findings of a Cross-Sectional Survey on Knowledge, Attitudes, and Practices about COVID-19 in Uganda: Implications for Public Health Prevention and Control Measures

**DOI:** 10.1155/2020/5917378

**Published:** 2020-12-04

**Authors:** Gerald Okello, Jonathan Izudi, Steven Teguzirigwa, Allan Kakinda, Guido Van Hal

**Affiliations:** ^1^Department of Epidemiology and Social Medicine, Faculty of Medicine and Health Sciences University of Antwerp, Belgium; ^2^Department of Community Health, Faculty of Medicine, Mbarara University of Science and Technology, Mbarara, Uganda

## Abstract

**Background:**

The coronavirus disease (COVID-19) morbidity is rising in Uganda. However, data are limited about people's knowledge, attitudes, and practices.

**Objective:**

To determine knowledge about COVID-19, attitudes towards presidential directives and Ministry of Health (MoH) guidelines, and adherence to practicing public health preventive measures (KAP) in Uganda.

**Methods:**

This cross-sectional survey was conducted between April 28 and May 19, 2020. Data were collected using online social media platforms, websites, and popular media outlets. We descriptively summarized data and categorized KAP scores as knowledgeable about COVID-19, positive attitude towards presidential directives and MoH guidelines, and adherent to public health preventive measures, respectively. We tested sex differences in KAP using tests of significance and established independently associated factors using modified Poisson regression analysis, reported using adjusted prevalence risk ratio (aPR) with 95% confidence interval (CI).

**Results:**

We studied 362 participants with the following sociodemographic characteristics: 86 (23.8%) aged 25-29 years, 212 (58.6%) males, 270 (74.6%) with tertiary or university levels of education, and 268 (74.0%) urban residents. Of the 362 participants, 264 (93.9%) were knowledgeable about COVID-19 (94.1% males and 93.8% females), 51.3% had positive attitudes towards presidential directives and MoH guidelines (51.0% male and 51.8% female), and 175 (48.3%) were adherent to practicing public health preventive measures (42.9% males and 56.0% females). Compared to males, our data shows that females were more adherent to practicing public health preventive measures (aPR, 1.23; 95% CI, 1.01-1.53), knowledgeable about COVID-19 (aPR, 1.01; 95% CI, 0.95-1.07), and had positive attitudes towards directives and guidelines (aPR, 1.01; 95% CI, 0.82-1.25).

**Conclusions:**

This study shows that public health prevention efforts should be directed to closing the identified gaps in KAP among Ugandans in order to halt the spread of COVD-19 in Uganda as well as the East African region.

## 1. Introduction

Currently, the world is experiencing the novel severe acute respiratory syndrome coronavirus disease 2019 (n-SARS-CoV-2) pandemic, commonly known as COVID-19, which was first reported by the World Health Organization (WHO) on December 31, 2019, as a viral pneumonia outbreak of unknown aetiology in the Hubei Province of China [1]. To date, at least eight million people are infected by COVID-19 and over 400,000 have died, with most counties in Europe worst hit by the pandemic [2]. COVID-19 is rapidly spreading across Africa, and current data indicates 54 countries are affected [3], with close to 200,000 people infected and deaths exceeding 4,000 [2]. Uganda, one of the countries in east Africa, reported its first case of COVID-19 on March 21, 2020, from international travels, and since then, the number of new infections has risen to over 700 as of June 18, 2010, largely driven by truck drivers from her neighboring countries [4].

Coronavirus is transmitted from person-to-person through droplets of saliva or discharge from the nose when an infected person coughs or sneezes [5–7]. Infected persons present with mild to moderate symptoms but are able to recover even without treatment [7]. The common symptoms include fever, tiredness, dry cough, shortness of breath, body aches and pains, and sore throat, and very few people present with diarrhoea, nausea, and running nose [7, 8]. Factors like old age and comorbidities, namely, cardiovascular diseases, diabetes mellitus, chronic respiratory diseases, and cancer, are associated with poor prognosis [7]. Without effective treatment and vaccine [9], the world is left with a single option thus strict adherence to public health preventive measures: regular handwashing using soap and water or alcohol-based hand rub, social distancing (maintaining a distance of at least two meters), not touching the face, covering the nose and mouth with tissue when coughing or sneezing, staying at home if feeling unwell, wearing of face masks, and prompt seeking of medical care when one has suggestive symptoms [7, 9, 10].

These measures have been popularized and supported by the WHO, governments, and Ministries of Health globally. To that effect, guidance and policies and presidential directives have been issued.

In Uganda, several communication channels are used to reach the population with preventive messages about COVID-19, including presidential directives. This is aimed at improving people's knowledge about COVID-19, changing their attitudes towards adopting public health preventive measures, and improving their adherence to practicing public health preventive measures. However, data are limited regarding people's knowledge about COVID-19, attitudes towards presidential directives and Ministry of Health (MoH) guidelines, and practices of public health preventive measures (KAP). Second, anecdotal observations indicate that males are less adherent to practicing public health preventive measures about COVID-19 compared to females, suggesting possible deficiency in knowledge about COVID-19 and perhaps negative attitudes towards presidential directives and MoH guidelines. However, evidence to support this observation is nonexistent. We therefore conducted a national study to primarily assess knowledge about COVID-19, attitudes towards presidential directives and Ministry of Health (MoH) guidelines, and adherence to practicing public health preventive measures (KAP) among Ugandans aged ≥ 18 years. Our secondary objective was to determine whether there are differences in KAP between males and females. We hypothesized that there is a difference in KAP according to sex. Our findings will inform the design of effective public health preventive measures so as to halt the spread of COVID-19.

## 2. Methods and Materials

### 2.1. Study Setting

This study was conducted in Uganda, a landlocked country, in east Africa. Uganda is bordered by Kenya in the east, South Sudan in the north, Democratic Republic of Congo in the west, and by Tanzania and Rwanda in the south. The total population of Uganda according to the 2014 census is 34,634,650 people, and 45% (15,585,593) are aged ≥ 18 years. Of those ≥18 years, 7.3% (1,137,749) have access to internet services [11]. Uganda is made up of 134 districts and 6,937 health facilities, of which 3,133 are government owned.

The distribution of health facilities is as follows: five national referral hospitals, 14 regional referral hospitals, 169 general hospitals, 194 health center (HC) IVs (a county level of health facility), and the rest are HC IIIs (subcounty level of health facilities) and HC IIs (parish level health facilities). There are five super specialized hospitals and two specialized institutes, the Uganda Heart Institute and Uganda Cancer Institute [12].

### 2.2. Study Design and Population

We conducted a cross-sectional study and the findings are reported in accordance to the guidelines of Strengthening of the Reporting of Observational Studies in Epidemiology (STROBE) [13, 14]. The study population consisted of Ugandans aged ≥ 18 years with access to online platforms like WhatsApp, Facebook, Twitter, and Instagram among others. Using Yamane's formula, 2.5% sampling error, and the number of people aged ≥ 18 years with access to internet services, we determined that 1,598 people would be needed. We approached 419 participants but 86.4% (*n* = 362) accepted to participate in the survey. Although online surveys are associated with low response rate, daily payments for over-the-top tax (OTT, a social media tax for online services) in order to access social media platforms and lockdown during the survey period led to restricted access to mobile data, and this was worsened by the closure of internet shops, and difficulties in accessing internet services in remote settings all contributed to the low sample size.

### 2.3. Data Collection and Measurements

Due to approximately three months of lockdown and restricted movements to minimize the spread of COVID-19, community-based national survey was not logistically feasible. Data were therefore collected between April 28 and May 19, 2020, using KoboToolbox, an online software that enabled the uploading of questionnaire online.

We utilized WhatsApp groups, Facebook, Twitter, Instagram, websites, and official accounts (Facebook, WhatsApp, and Twitter) of local popular media outlets, which are the available online platforms in the country for data collection and to achieve maximum coverage. We repeatedly provided reminders after every 2 days to improve participant response rate. The questionnaire had questions about KAP and was developed using the Uganda MoH and the World Health Organization (WHO) guidelines on prevention of COVID-19. To arouse participants' interest in taking the survey, we developed one-page recruitment poster with a link to the online questionnaire that contained brief information about the study objectives, importance, and ethical concerns. Once the poster was filled and the individual was eligible, access to the questionnaire was automated.

We collected data on participants' age measured in years and later categorized into several age groups, namely, 18-24, 25-29, 30-34, 35-39, 40-44, and ≥45 years; sex measured as male or female; marital status measured as never married/single or married/staying with partner; level of education measured as none/never received formal education, primary, secondary, and tertiary/university education; current occupation measured as unemployed, self-employed, and formal employment; religion measured as Anglican, Catholic, Muslim, Pentecostal, and others (Seventh day Adventists (SDA), Jehovah Witness, Orthodox, Hindu, Humanist, and Bahai); district of residence that consisted of the 134 districts in Uganda, and participant residence measured as urban/periurban or rural. We categorized the districts of residence into 5 regions: central, eastern, Kampala, northern, and western.

We assessed the knowledge scores using 16-item Likert scale questions coded 1-3 to denote true, false, and do not know. The 16 questions were about the signs and symptoms of COVID-19, transmission, mode of spread, prevention, and treatment. For attitude scores, we used five questions, each scored on a scale of 1-5, with the lowest score being strongly disagree and the highest as strongly agree. The five questions focused on the WHO and Uganda MoH guidelines for the prevention of COVID-19, presidential directives, effectiveness of preventive measures that are in use, an assessment of the success of preventive measures, and adequacy of different communication channels.

We used 10 questions with measurements on binary scale (yes or no) to measure practices of public health preventive measures. The questions focused on the frequency of handwashing, staying at home for at least five hours, wearing of gloves and masks whenever leaving home, avoidance of handshaking and crowded places, and promptness in seeking treatment in the event of signs and symptoms suggestive of COVID-19 and notification of relevant authorities like the local council system, police, and health authorities.

### 2.4. Quality Control Measures

To determine the appropriateness, logical flow, and consistency of questions in the questionnaire, we conducted an online pretest in the neighboring country, Kenya. The respondents provided comments on the logical flow, understanding, and relevance of the questions. We used the comments to revise and to develop the final questionnaire. During data collection, we integrated quality control measures to ensure participation of eligible individuals only. We also used a unique password to protect filled questionnaires and restrict data access to the data analyst. We used VeraCrypt, an open source encryption software, to share data among the research team for the purposes of validation. At data analysis stage, we checked for data consistency, cleaned, and transformed variables.

### 2.5. Statistical Analysis

We descriptively summarized categorical data using frequencies and percentages and numerical data using means with standard deviations or medians with interquartile ranges (IQR). For KAP studies, Bloom recommends the following cutoff points: (1) 80-100% for high knowledge, positive attitude, and good practice; (2) 60-79% for moderate knowledge, neutral attitude, and fair practice; and (3) less or equals 59% for low knowledge, negative attitude, and poor practice [15, 16]. In this KAP study, we used a cutoff of 75%, which was a modification of the Bloom's cutoff point.

Accordingly, we considered participants with scores ≥ 75% as knowledgeable about COVID-19, having positive attitude towards presidential directives and MoH guidelines, and adherent to public health preventive measures. Conversely, participants with scores < 75% were considered nonknowledgeable about COVID-19, having negative attitudes towards presidential directives and MoH guidelines, and nonadherent to public health preventive measures. We tested median differences in KAP scores with respect to sex using the two sample Wilcoxon's test at bivariate analysis. Furthermore, we assessed differences in proportions of KAP using the chi-square test for large cell counts (typically ≥5) and the Fisher's exact test for smaller cell counts (typically <5).

Variables with two-sided probability values less than 5% (*p* < 0.05) at bivariate analysis and those deemed biologically plausible for differences in KAP between males and females, namely, level of education, residence, and employment status were considered significant for multivariable analysis. We did not use binary logistic regression analysis because the outcomes were large and the use of odds ratio (OR) would overestimate the degree of association. Accordingly, prevalence risk ratios (PRs) were computed using a modified Poisson regression with robust standard errors to control for mild violations of the assumptions [17–19]. We reported each PR with the corresponding 95% confidence interval (CI). This analysis was performed in Stata version 15 [20].

### 2.6. Ethical Considerations

We obtained informed consent using an informed consent form (ICF) attached to the online questionnaire. We ensured that the questionnaire was inaccessible without filling the ICF that described the potential benefits and risks and rationale for the survey. The ICF mentioned that participation was voluntary, and withdrawal was allowable at any stage. Ethical review and approval was obtained from the AIDS Support Organization Research Ethics Committee (TASO-REC), and reference number is TASOREC/032/2020-UG-REC-009.

## 3. Results

### 3.1. Sociodemographic Characteristics of Respondents

The mean age of the 362 participants was 33.5 ± 10.4 years, with a median age of 31 years (IQR: 18-75). 212 (58.6%) participants were males, another 212 (58.6%) were married or staying with the partner at the time of the survey, 270 (74.6%) had attained tertiary or university levels of education, 268 (74.0%) were rural residents, and 102 (28.2%) were residents of Kampala district. Furthermore, almost half of the participants (48.9%) had formal employment, and 43.9% were of the Catholic religion (Table 1).

### 3.2. Sources of Information about COVID-19 in Uganda

The predominant source of information was television (77.4%), and the least was the local council (LC) system at 11.0%. More males were reached with information via television than females: 80.7% versus 72.7%, *p* = 0.073. 265 (73.2%) reported the social media as the commonest source of information, with more males having access (76.9%) compared to females (68.0%), *p* = 0.060. 280 (68.5%) reported the radio, with many of them being males than females: 70.3% versus 66.0%, *p* = 0.389. 161 (44.5%) reported short messaging signals (SMS), with 101 (47.6%) being males and 60 (40.0%) females, *p* = 0.150. The other sources of information are summarized in Supplementary Table 1.

### 3.3. Distribution of KAP Level by Sociodemographic Characteristics

Table 2 summarizes the distribution of participants who were found knowledgeable about COVID-19, had positive attitudes towards presidential directives and MoH guidelines, and were adherent to public health preventive measures. Majority of the participants, 93.9% (94.1% males and 93.8% females), were knowledgeable about COVID-19. Table 2 further shows that the most knowledgeable participants were aged 35-39 years (97.4%), male (94.1%), married or staying with a partner (94.7%), had reached secondary level of education (100.0%), urban (94.6%) and Kampala (96.5%) residents, had formal employment (95.9%), and were those affiliated to Pentecostal (97.5%).

We found no differences in proportion of knowledge about COVID-19 with respect to sociodemographic characteristics. Overall, only half of the respondents (51.3%) had positive attitudes towards the presidential directives and MoH guidelines.

Most participants with positive attitudes about the presidential directives and MoH guidelines were aged ≥ 45 years (62.5%), female (51.8%), married or staying with the partner (52.3%), had reached at least primary level of education (73.3%), rural (58.4%) and Eastern residents (66.1%), respectively, self-employed (57.4%), and Catholics (56.9%) as shown in Table 2. We observed statistically significant differences in proportion of positive attitudes towards presidential directives and MoH guidelines with respect to level of education (*p* = 0.019).

Despite the high level of knowledge about COVID-19 among the participants, overall, less than half of the participants (48.3%) were adherent to practicing the MoH COVID-19 public health preventive measures (Table 2). Results further indicated that the most adherent participants were mainly aged ≥ 45 years (61.2%), female (56.0%), married or staying together (48.6%), had at least secondary level of education (71.4%), rural residents (50.0%), living in Western region (81.3%), self-employed (57.3%), and Anglicans (53.0%). Statistically significant differences in adherence to presidential directives and MoH guidelines were observed in sex (*p* = 0.014), level of education (*p* = 0.03), region (*p* < 0.001), and employment (*p* = 0.022). Elsewhere (supplementary tables), specific proportions of scores on knowledge about COVID-19 (Supplementary Table 2), attitudes towards directives and guidelines (Supplementary Table 3), and practices of public health preventive measures (Supplementary Table 3) are summarized.

### 3.4. Median Differences in KAP Scores between Males and Females in Uganda

Table 3 summarizes the median KAP scores. The median knowledge score was 13 (IQR: 12-14). Although the average knowledge score was higher among males than females, the observed difference was not statistically significant: 13 (IQR: 12-14) versus 13 (IQR: 12-13), *p* = 0.258. The median attitude score was 22 (IQR: 19-24). Again, there was no statistically significant difference in attitude scores between males and females: 22 (IQR: 19-24) versus 22 (IQR: 19-24), *p* = 0.583. The median practice score was 7 (IQR: 7-8), with no statistically significant differences between males and females: 7 (IQR: 7-8) versus 8 (IQR: 7-9), *p* = 0.095.

### 3.5. Analysis of Sex Differences in KAP between Males and Females

In the unadjusted analysis (Table 4), compared to males, females were more likely to have positive attitudes towards presidential directives and MoH guidelines (PR, 1.01; 95% CI, 0.82-1.25) and more adherent to practicing recommended public health preventive measures (PR, 1.30; 95% CI, 1.06-1.61). In the adjusted analysis, results showed that females were not significantly more knowledgeable about COVID-19 (aPR, 1.01; 95% CI, 0.95-1.07) or had more positive attitudes towards presidential directives and MoH guidelines (aPR, 1.01; 95% CI, 0.82-1.25) than males. However, they were 23% more likely to practice recommended public health preventive measures than males (aPR, 1.23; 95% CI, 1.01-1.53).

## 4. Discussion

This is the first national cross-sectional survey to assess knowledge of Ugandans about COVID-19, their attitudes towards presidential directives and MoH guidelines, and adherence to practicing public health preventive measures. Our data shows that approximately 94% of Ugandans are knowledgeable about COVID-19, and almost 50% had positive attitudes towards presidential directives and MoH guidelines, and less than one in every two are adherent to practicing the recommended public health preventive measures. Most participants knew the main symptoms of COVID-19, mode of transmission, high-risk groups and that there is no effective treatment or vaccine against COVID-19, the importance of supportive treatment, and public health preventive measures, namely, handwashing, personal respiratory hygiene, wearing of masks in public, and isolation areas. Our findings are in agreement with studies conducted in Iran, Tanzania, Paraguay, Malaysia, and China that all show high knowledge scores regarding COVID-19 [21–23]. Nonetheless, our findings differ from studies conducted in Bangladesh and Malaysia that show an overall low knowledge score [24, 25]. Although we are not certain about the implementation of public health preventive approaches in those countries with low knowledge scores, in Uganda, the dissemination of public health messages trickles up to the lowest administrative level. This might explain the high knowledge scores observed in the study. We found no difference in knowledge scores by sociodemographic characteristics, namely, sex, age, education, and residence among others, contrary to findings in Bangladesh [25], Tanzania [21], and Iran [22], possibly due to constant presidential directives and widespread dissemination of MoH guidelines.

The main sources of information about COVID-19 included televisions, social media, radios, and short text messages signals (SMS), perhaps because most of the participants were urban residents, literate, and had formal employment. COVID-19 information could have easily reached the participants via online media, television, and radio and MoH directed text messages.

Our study shows that participants have trust in the presidential directives and MoH guidelines, with most reporting that the directives and guidelines are adequate and necessary to halt the spread of COVID-19. Besides, participants have confidence that the country is on course in winning the battle against COVID-19, which is similar to earlier findings in China [26]. This trust and confidence might have resulted from the numerous immediate steps like ban on international and regional travels, curfews, and lockdown among others that were implemented in the country to combat the spread of COVID-19 following the confirmation of the first case on March 21, 2019. However, the overall attitude towards the presidential directives and MoH guidelines is low, and this might translate to compromised adherence to practicing public health preventive measures, which might pose public health threat of community transmission of COVID-19. Our findings differ from studies conducted in China, Tanzania, Paraguay, and Iran that found high attitude scores [21, 23]. In our study, the attitude scores are different with respect to levels of education, which is similar to findings in Iran [22].

Our data show low adherence to public health preventive measures, which is not surprising because this study found low score on attitude towards presidential directives and MoH guidelines. Although our overall finding is contrary to earlier results in Iran [22], the findings on specific practices were in agreement with several studies elsewhere [21, 23, 24]. Furthermore, our data show that adherence to recommended public health preventive measures significantly vary with respect to sex, level of education, age, region, and employment status, which is consistent with a study in Iran [22]. In particular, our study shows that females are more adherent to public health preventive measures than males, suggesting sex-specific measures might be useful in promoting adherence to public health preventive measures and consequently in combatting the spread of COVID-19 in the country.

### 4.1. Study Strengths and Limitations

This is the first study in Uganda to determine knowledge about COVID-19, attitudes towards presidential directives and MoH guidelines, and adherence to practicing public health preventive measures. This study covered all the regions in Uganda and thus presents credible evidence that can inform the Uganda MoH, policy makers, epidemiologists, public health practitioners, health service managers, and researchers among others about prospective public health information dissemination strategies, as well as existing gaps that need immediate public health preventive measures.

Despite these strengths, there are numerous limitations that should be considered in the interpretation of the results. Our sample size was relatively small compared to what we had desired despite repeated posting of online survey questionnaires. This was not surprising because during the data collection period, the government of Uganda had instituted curfews, restricted movements to critical workers particularly security personnel and healthcare providers, and all shops that were selling nonfood items where potential participants would buy airtime in order to have mobile data to enable filling of the questionnaire were closed. Second, since payment of OTT is compulsory in the country, this might have been a deterrent factor in accessing social media platforms, our main data collection channel. Moreover, we did not provide financial support to encourage data access to the social media platforms used for data collection. Therefore, our responses are limited to those who were able to pay in order to access these platforms. However, the data on KAP were uniformly distributed suggesting that the present sample size is sufficient for statistical inference. Since this was a cross-sectional study, our findings demonstrate an association and with no temporal relationship. Our study had more residents from urban areas and Kampala region and people with relatively high levels of education. The findings might not therefore reflect KAP scores in rural areas and among the illiterate subgroups of the population. However, we attempted to minimize this limitation through adjusted analysis. Even with adjusted analysis, we acknowledge that residual confounding is possible. Also, since practice of public health preventive measures was a self-reported outcome, social desirability bias remains a possibility.

Lastly, we did not study several factors which contribute to differences in KAP between males and females such as sources, access, frequency and intensity of exposure to health information including cultural differences among others. We recommend that prospective studies should consider these factors.

### 4.2. Conclusions and Recommendations

Our data show a high proportion of knowledge about COVID-19 and relatively low positive attitudes towards presidential directives and MoH guidelines as well as low adherence to practices of public health preventive measures. We observed sex differences in practicing the recommended public health preventive measures, with females being more dominant than males. We found no sex differences in knowledge about COVID-19 and attitudes towards presidential directives and MOH guidelines. We conclude that the current public health preventive efforts should be directed towards closing the identified gaps in KAP. This will help to halt the spread of COVID-19 in Uganda and the East African region.

## Figures and Tables

**Table 1:** Sociodemographic characteristics of the participants.

Characteristics	Level	Frequency (%)
Age group (years)	18-24	65 (18.0)
	25-29	86 (23.8)
	30-34	77 (21.2)
	35-39	48 (13.3)
	40-44	37 (10.2)
	≥45	49 (13.5)
	Mean (SD)	33.5(10.4)
Sex	Male	212 (58.6)
	Female	150 (41.4)
Marital status	Never married	150 (41.4)
	Married/staying with partner	212 (58.6)
Education	None	3 (0.8)
	Primary	40 (11.1)
	Secondary	49 (13.5)
	Tertiary/university	270 (74.6)
Residence	Urban	268 (74.0)
	Rural	94 (26.0)
Region	Central	99 (27.4)
	Eastern	62 (17.1)
	Kampala	102 (28.2)
	Northern	67 (18.5)
	Western	32 (8.8)
Employment	Unemployed	78 (22.1)
	Self-employed	103 (29.2)
	Formal employment	172 (48.7)
Religion	Anglican	100 (27.6)
	Catholic	156 (43.9)
	Muslim	35 (9.7)
	Pentecostal	51 (14.1)
	Others	17 (4.7)

**Table 2:** Distribution of KAP level by sociodemographic characteristics.

Outcomes	Had knowledge about COVID-19 (*n* = 264)		Had positive attitudes towards presidential directives and MoH guidelines (*n* = 175)		Adherent to practicing public health preventive measures (*n* = 175)	
Characteristic	No. (%)	*p* value	No. (%)	*p* value	No. (%)	*p* value
Age group in years						
18-24	35 (87.5)	0.503	32 (51.6)	0.653	33 (50.8)	0.059
25-29	62 (93.9)		43 (51.8)		48 (55.8)	
30-34	61 (93.8)		39 (51.3)		32 (41.6)	
35-39	37 (97.4)		20 (44.4)		18 (37.5)	
40-44	33 (97.1)		16 (45.7)		14 (37.8)	
≥45	36 (94.7)		25 (62.5)		30 (61.2)	
Sex						
Male	158 (94.1)	0.933	103 (51.0)	0.883	91 (42.9)	0.014
Female	113 (93.8)		72 (51.8)		84 (56.0)	
Marital status						
Never married	102 (92.7)	0.490	73 (50.0)	0.673	72 (48.0)	0.913
Married/staying with partner	162 (94.7)		102 (52.3)		103 (48.6)	
Education level						
None	1 (100.0)	0.213	1 (33.3)	0.019	1 (33.3)	0.003
Primary	19 (86.4)		22 (73.3)		22 (55.0)	
Secondary	34 (100.0)		24 (63.3)		35 (71.4)	
Tertiary/university	210 (93.7)		128 (47.4)		117 (43.3)	
Residence						
Urban	208 (94.6)	0.427	130 (49.2)	0.155	128 (41.8)	0.709
Rural	56 (91.8)		45 (58.4)		47 (50.0)	
Region						
Central	72 (92.3)	0.684	48 (48.5)	0.097	44 (44.4)	<0.001
Eastern	43 (93.5)		41 (66.1)		30 (48.4)	
Kampala	82 (96.5)		48 (47.1)		52 (51.0)	
Northern	40 (95.2)		31 (46.3)		23 (34.3)	
Western	27 (90.0)		7 (63.6)		26 (81.3)	
Employment status						
Unemployed	52 (94.6)	0.281	35 (51.5)	0.312	42 (53.8)	0.022
Self-employed	67 (90.5)		54 (57.4)		59 (57.3)	
Formally employed	139 (95.9)		81 (47.6)		71 (41.3)	
Religious affiliation						
Anglican	78 (90.7)	0.256	47 (50.5)	0.302	53 (53.0)	0.569
Catholic	111(95.7)		87 (56.9)		75 (47.2)	
Muslim	25 (96.1)		15 (46.9)		18 (51.4)	
Pentecostal	39 (97.5)		20 (40.0)		20 (39.2)	
Others^#^	11 (84.6)		6 (46.1)		9 (52.9)	

Note: values are row percentages expressed as *n*. ^#^Includes Seventh day Adventists (SDA), Jehovah Witness, Orthodox, Hindu, Humanist, and Bahai.

**Table 3:** Median differences in KAP scores between males and females in Uganda.

	Overall	Male	Female	*p* value
	Median score (IQR)	Median score (IQR)	Median score (IQR)	
Knowledge about COVID-19	13 (12-14)	13 (12-14)	13 (12-14)	0.258
Attitude towards presidential directives and MoH guidelines	22 (19-24)	22 (19-24)	22 (19-24)	0.583
Practices public health preventive measures	7 (7-8)	7 (7-8)	8 (7-9)	0.095

**Table 4:** Unadjusted and adjusted analyses of sex differences in KAP between males and females.

Characteristics	Knowledgeable about COVID-19		Had positive attitudes towards presidential directives and MoH guidelines		Adherent to practicing public health preventive measures	
	PR	aPR	PR	aPR	PR	aPR
Male	1	1	1	1	1	1
Female	1.00 (0.94-1.06)	1.01 (0.95-1.07)	1.01 (0.82-1.25)	1.01 (0.82-1.25)	1.30 (1.06-1.61)^∗^	1.23 (1.01-1.53)^∗^

Note: (1) ^∗^*p* < 0.05; ^∗∗∗^*p* < 0.001; ^∗∗∗^*p* < 0.0001 at 5% significance level; (2) prevalence risk ratios (PRs) are exponentiated with confidence intervals in brackets; (3) all aPRs were adjusted for level of education, residence, and employment; (4) PR: unadjusted prevalence risk ratio; (5) aPR: adjusted prevalence risk ratio; (6) PRs were computed with males as the comparison group.

## Data Availability

The data used/analyzed in this study are available on reasonable request from the corresponding author.

## Supplementary Materials

Supplementary Materials Supplementary Table 1. Sources of information about COVID-19 in Uganda. Supplementary Table 2. Distribution of knowledge by sex. Supplementary Table 3. Distribution of attitude by sex. Supplementary Table 4. Distribution of practices by sex.
