# Supplementary material for: Findings of a Cross-Sectional Survey on Knowledge, Attitudes, and Practices about COVID-19 in Uganda: Implications for Public Health Prevention and Control Measures
Source: Biomed Res Int. 2020 Dec 4;2020:5917378. doi: 10.1155/2020/5917378 (PMC7729389; doi:10.1155/2020/5917378)
Supplement: Supplementary Materials — Supplementary Table 1. Sources of information about COVID-19 in Uganda. Supplementary Table 2. Distribution of knowledge by sex. Supplementary Table 3. Distribution of attitude by sex. Supplementary Table 4. Distribution of practices by sex. [file 5917378.f1.docx]

**Supplementary Table 1: Sources of information about COVID-19 in Uganda**

| **Source of information** | **Total, no. (%)** | **Male, no. (%)** | **Female, no. (%)** | **P value** |
| --- | --- | --- | --- | --- |
| Television | 280 (77.3) | 171 (80.7) | 109 (72.7) | 0.073 |
| Social media | 265 (73.2) | 163 (76.9) | 102 (68.0) | 0.060 |
| Radio | 248 (68.5) | 149 (70.3) | 99 (66.0) | 0.389 |
| Short text message signals | 161 (44.5) | 101 (47.6) | 60 (40.0) | 0.150 |
| Leaflets of Uganda MoH | 72 (19.9) | 48 (22.6) | 24 (16.0) | 0.119 |
| Local Council | 40 (11.0) | 28 (13.2) | 12 (8.0) | 0.120 |
| News paper | 42 (11.6) | 31 (14.6) | 11 (7.3) | 0.033 |
| Others | 79 (21.8) | 58 (27.4) | 21 (14.0) | 0.002 |
